# Relationship of Autophagic Dysfunction With the Quality of Life and Sleep, Depression and Disease Severity in Patients With Restless Legs Syndrome

**DOI:** 10.1002/brb3.70172

**Published:** 2025-01-07

**Authors:** Esen Çiçekli, Hamza Malik Okuyan, Canan Birimoğlu Okuyan, Şeyda Öznur Ayçiçek, Dilcan Kotan

**Affiliations:** ^1^ Department of Neurology Akyazı State Hospital Sakarya Türkiye; ^2^ Faculty of Health Sciences, Department of Physiotherapy and Rehabilitation, Biomedical Technologies Application and Research Center, Physiotherapy and Rehabilitation Application and Research Center Sakarya University of Applied Sciences Sakarya Türkiye; ^3^ Faculty of Health Sciences Department of Nursing Sakarya University of Applied Sciences Sakarya Türkiye; ^4^ Faculty of Medicine, Department of Neurology Sakarya University Sakarya Türkiye

**Keywords:** ATG3, ATG5, autophagy, diagnostic marker, restless leg syndrome

## Abstract

**Introduction:**

Restless legs syndrome (RLS) is a frequently encountered neurological illness that has no effective treatment and imposes an enormous socioeconomic burden. Autophagy is essential for the maintenance of healthy cellular physiology, cell viability, and defense against pathogenic conditions. However, there is no study investigating the possible role of autophagy‐related proteins (ATGs) in RLS patients. Therefore, we aimed to investigate the expression and diagnostic potential of ATG3 and ATG5, as well as the relationships between these proteins and laboratory markers, depression, disease score, quality of life, and sleep in RLS patients.

**Methods:**

A total of 49 patients with RLS and 26 healthy individuals were recruited for the current study. The severity of the disease was assessed using the international RLS rating scale. All participants were administered the Pittsburgh Sleep Quality Index, the Quality‐of‐Life Scale, and the Beck Depression Inventory. The enzyme‐linked immunosorbent assay was employed to quantify the expressions of ATG3 and ATG5.

**Results:**

Serum ATG3 and ATG5 expressions were significantly upregulated in RLS patients compared to healthy controls (*p* = 0.005) and upregulated ATG3 and ATG5 expressions were relationship with the severity of the disease (*p* < 0.05). ATG3 was substantially correlated with the quality of sleep, whereas ATG5 was correlated with the quality of life and depression status (*p* < 0.05). The ROC curve analysis demonstrated that ATG3 expressions over 3146.5 ng/mL and ATG5 expressions over 4732.5 ng/mL may predict the presence of RLS (*p* < 0.01).

**Conclusion:**

We report for the first time that autophagy may have a significant impact on the development of RLS.

## Graphical abstract

In summary, autophagy may play a crucial role in the development of Restless Legs Syndrome (RLS). Autophagic dysfunction is associated with a worse quality of life, disturbed sleep, and the severity of the disease in patients with RLS. Autophagy‐related Protein 3 (ATG3) and Autophagy‐related Protein 5 (ATG5) may serve as prognostic or diagnostic markers for RLS. Upregulated ATG5 expressions are associated with depression status in RLS.

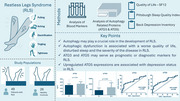

## 1 Introduction

Restless legs syndrome (RLS) is a prevalent neurological disease that has a detrimental impact on individuals' quality of life and sleep (Gossard et al. 2021). The frequency of RLS ranges from 1% to 20% across various groups (Allen et al. 2003; Yeh, Walters, and Tsuang 2012). Additionally, RLS is linked to several clinical disorders, and there is compelling data indicating that the occurrence of RLS rises notably in clinical situations such as epilepsy, Parkinson's disease (PD), pregnancy, diabetes, migraine and renal diseases (Maggi et al. 2024; Talaia et al. 2024; Trenkwalder et al. 2016). The epidemiological research on RLS confirms that it is more common in certain neurological disorders (Ghasemi et al. 2020; Maggi et al. 2024; Talaia et al. 2024). For instance, approximately 15 out of every 100 adults with epilepsy experience symptoms of RLS (Talaia et al. 2024). Furthermore, PD patients have a higher incidence of RLS than the general population (Maggi et al. 2024). RLS was present in 16.3% of migraine sufferers overall (Ghasemi et al. 2020). These findings demonstrate how serious a health issue RLS is for the general public. The occurrence of the illness is linked to both the family's medical background and genetic predisposition (Högl et al. 2005). Recent research indicates that reduced iron levels in the central nervous system and impaired dopaminergic function are significant factors in the development of RLS (Allen 2007; Weinstock, Walters, and Paueksakon 2012). Research on the nervous system reveals that individuals with RLS have low levels of iron in their brains (Allen et al. 2001; Connor et al. 2003). Nevertheless, the exact cause of this deficiency is not yet completely understood. Research has demonstrated that the cerebrospinal fluids of individuals with early‐stage RLS exhibit an increase in transferrin levels and a decrease in ferritin levels (Connor et al. 2003; Mizuno et al. 2005). Iron is an essential metal that acts as a cofactor for tyrosine hydroxylase, an enzyme necessary for the production of dopamine, and is crucial for maintaining normal cellular processes (Cho, Kim, and Lee 2017). Normal physiological function of a cell depends on a well‐controlled iron balance. Because of this, iron deficiency—which is often seen in RLS patients—is believed to be effective throughout the syndrome's progression (Allen 2007). Iron deficiency, ferritin dysfunction and dopaminergic dysfunction have been extensively examined in studies aimed at understanding the molecular pathophysiology of RLS (Nanayakkara, Di Michiel, and Yee 2023). Furthermore, there is evidence that other possible cellular processes associated with these mechanisms, such as autophagy, may play an active role in the pathogenesis of RLS (Inoue et al. 2017; Qin et al. 2021).

Autophagy is a protective molecular mechanism that contributes to cellular homeostasis by degrading and recycling intracellular components, including protein aggregates and damaged organelles (Liu et al. 2023). Autophagy plays a key function in healthy cellular physiology, cell viability, and defense against pathogenic conditions, but its dysregulation has been linked to a variety of disorders, including neurodegeneration, infection and cancer (Arroyo et al. 2014; Okuyan et al. 2021; Xu et al. 2019). Dysregulated autophagy is associated with an iron shortage, which exacerbates RLS symptoms (Inoue et al. 2017; Krishan et al. 2015; Nanayakkara, Di Michiel, and Yee 2023). Moreover, dysfunction of the dopaminergic system, which is responsible for the development of RLS, can impair the process of autophagy (Munoz et al. 2012; Zhang et al. 2021). Hence, autophagy may be a critical factor in the development of RLS. Moreover, the growing body of evidence indicates that altered levels of autophagy‐related proteins in the blood may serve as diagnostic or prognostic markers for several diseases, such as Alzheimer's disease, multiple sclerosis, Coronavirus disease 2019, gastric cancer, and malignant mesothelioma (Cao et al. 2016; Cho et al. 2019; Joodi Khanghah et al. 2020; Okuyan et al. 2021; Tomasetti et al. 2023).

Autophagy‐related gene 3 (ATG3) is a protein that plays a role in the process of LC3 lipidation, which is essential for the formation of autophagy (da Silva Lima et al. 2022). ATG3 has received comparatively little research attention in comparison to other autophagic proteins. Experimental studies have demonstrated that ATG3 plays a pivotal role in neurological illnesses, such as Parkinson's disease and ischemic cerebral stroke, by regulating cell damage, inflammation, and cell death (Dong et al. 2024; Peng et al. 2021). Moreover, it has been shown that ATG3 may have prognostic and diagnostic worth for some disorders. For example, ATG3 could be employed as a gastric cancer predictive biomarker (Cao et al. 2016). Autophagy‐related gene 5 (ATG5) is a necessary regulator protein of the autophagy system, responsible for controlling the creation of autophagosomes at the most critical step (Changotra et al. 2022). In addition, ATG plays a crucial role in both physiological and pathological conditions, and a wide range of illnesses have shown dysregulation of this protein (Changotra et al. 2022). Previous research has revealed that ATG5 may have diagnostic value for a variety of disorders, including Parkinson's disease, Alzheimer's disease, multiple sclerosis, and allergic rhinitis (Cho et al. 2019; Joodi Khanghah et al. 2020; Zheng and Wang 2023). Though the diagnostic and therapeutic implications of autophagic proteins, like ATG3 and ATG5, in various human diseases have lately been better appreciated, their potential role in RLS remains uncertain.

Considering the aforementioned functions of autophagic proteins in various pathological conditions, we hypothesized that ATG3 and ATG5 might have diagnostic and therapeutic implications for the treatment of RLS. Thus, we first aimed to investigate the expression of autophagy‐related proteins in patients with RLS and its diagnostic potential for effective clinical management of RLS. Second, we investigated if these proteins were associated with certain laboratory markers, depression, illness score, quality of life, and sleep in RLS patients.

## 2 Material and Methods

### 2.1 Subjects

The study included a total of 49 patients who received therapy at the Neurology outpatient clinics of Sakarya Training and Research Hospital and Sakarya Akyazı State Hospital, and the study period spanned from December 2023 to April 2024. The patients were diagnosed with RLS by a physician, according to the criteria established by the International Restless Legs Syndrome Study Group (IRLSSG) (Allen et al. 2014). In addition, 26 individuals who gave their consent to participate in the research but had no medical diagnosis were added as control subjects. The current study was approved by Sakarya University's Clinical Research Ethics Committee (2023/11‐01) and we obtained informed consent from each participant prior to the project. Demographic information and clinical observations of all individuals were documented.

### 2.2 Inclusion and Exclusion Criteria

The following were the study's inclusion criteria: Greater than eighteen years of age, having a primary RLS diagnosis based on IRLSSG criteria in the patient group, exhibit normal results on neurological examination, and have no history of RLS in the healthy control group. The following were the exclusion criteria for all subjects in the study: people whose ferritin level has consistently been below 15 mg/L over the last three months (as per the Turkish Hematology Association's adult iron deficiency diagnosis and treatment guidelines); people who are pregnant; people who have an acute infection; people who have a neurological or chronic diseases (Parkinson's disease, multiple sclerosis, polyneuropathies, chronic renal failure, hepatic and hematological illnesses) that may cause secondary RLS. Further, to exclude polyneuropathy, nerve conduction studies were performed on all individuals in the patient group, and those with abnormal results were excluded in our study. The study included patients with RLS who took supplements such as iron, magnesium and B12.

### 2.3 Assessment of the Severity of RLS

According the international RLS rating scale (IRLSS), patients divided into four groups (Walters et al. 2014). We performed the assessment of the clinical severity in patients with RLS based on the criteria established by the IRLSSG and utilized the IRLSSG score to classify the severity of RLS (Walters et al. 2014). A score of less than 10 indicates mild RLS, a score between 10 and 20 indicates moderate RLS, a score between 20 and 30 indicates severe RLS, and a score greater than 30 indicates extremely severe RLS.

### 2.4 Assessment of Patients' Quality of Life

We evaluated patients' quality of life using SF‐12, a condensed and practical version of SF‐36 (Ware, Kosinski, and Keller 1996). Research on the test's reliability and validity in Turkey was carried out by Soylu and Kütük (2022). The SF‐12 scale assesses how health affects a person's everyday life. It consists of two scores: the physical component summary score (SF‐PCS12) and the mental component summary score (SF‐MCS12). Higher scores indicate better health; SF‐PCS12 and SF‐MCS12 ratings run from 0 to 100.

### 2.5 Evaluation of Sleep Quality

We evaluated the sleep quality and sleep disturbance of the participants for a duration of one month using the Pittsburgh Sleep Quality Index (PSQI). The PSQI scale, which was developed by Buysse et al. (1989), was translated into Turkish by Ağargün, Kara, and Anlar (1996). It assesses the quality of sleep and the disturbances experienced in the previous month and consists of 24 questions. The total score of the scale is determined by the sum of the scores of the seven components: Subjective Sleep Quality, Sleep Latency, Sleep Duration, Habitual Sleep Efficiency, Sleep Disturbance, Use of Sleeping Pills, and Daytime Dysfunction. A cumulative score exceeding 5 indicates “poor sleep quality.”

### 2.6 Evaluation of Depression in Patients With RLS

We assessed the depression status of patients with Restless Legs Syndrome (RLS) using the Beck Depression Inventory (BDI) designed by Beck in 1961. The Beck Depression Index (BDI) is a self‐notification scale measuring emotional, cognitive, physical, and motivating elements (Beck et al. 1961). Teğin (1987) and Hisli (1989) studied its validity and reliability in Türkiye. The scale consists of 21 items. Each of the 21 items was scored on a Likert scale ranging from 0 to 3. The 21‐item scale, categorizes mild depression as scoring between 14–19 points, whereas severe depression is indicated by scores between 20 and 63 points.

### 2.7 Blood Sample Collection and Blood Marker Analyses

We employed the vacuum tube technique to extract blood samples from all subjects, which were subsequently transferred into suitable laboratory tubes. Then, we allowed the blood samples to coagulate for around 20–30 min at room temperature in order to get serum samples and every blood sample was centrifuged at 1500 × *g* for 10 min at +4°C. Routine biochemical techniques were used to analyze hematological markers (white blood cells [WBC], red blood cells [RBC], hemoglobin [HGB], hematocrit [HCT], neutrophil [NEU]), four main electrolytes (sodium [Na], potassium [K], magnesium [Mg], and calcium [Ca]) and biochemical parameters (ferritin, iron, total iron binding capacity [TIBC], alanine aminotransferase [ALT], aspartate aminotransferase [AST], and creatinine [CREA]). The remain serum samples were then kept at –80°C until the Enzyme‐linked Immunosorbent Assay.

### 2.8 Analysis of Autophagic Proteins

Serum levels of ATG3 and ATG5 in patients with RLS were analyzed using ELISA. We used commercial kits for the ELISA to analyses the levels of autophagic proteins. The ATG3 ELISA kit has a detection range of 0.156–10 ng/mL, and the tested ATG3 protein exhibited intra‐ and inter‐assay coefficients of variance (CV%) below 7%. The sensitivity for the ATG3 measure is 0.094 ng/mL (Finetest, EH6526). Within the 0.313–20 ng/mL detection range of the ATG5 ELISA kit, the tested protein showed intra‐ and inter‐assay coefficients of variation (CV%) that were less than 7%. The ATG5 measure has a sensitivity of 0.188 ng/mL (Finetest, EH1729).

### 2.9 Statistical Analyses

We utilized either the Shapiro–Wilk or the Kolmogorov–Smirnov tests in order to investigate the normality distribution of the statistical data. After that, the Kruskal–Wallis *H* test followed by the Mann–Whitney *U* test was utilized to investigate the data that was not normally distributed, while the one‐way analysis of variance (ANOVA) test was utilized to analyze the data that appeared to be normally distributed. For the purpose of conducting correlation studies, we utilized either the Pearson's correlation or the Spearman's rho test, depending on which method was more appropriate. Whereas continuous variables were given as means and standard deviations, categorical data were represented as numbers and percentages. Moreover, to examine the diagnostic efficacy of autophagic proteins in the RLS screening, we carried out a receiver‐operating characteristic curve (ROC) analysis. We used the SPSS program version 23.0 (IBM Corporation, Armonk, NY, USA) and *p* values less than 0.05 was considered to indicate statistical significance.

## 3 Results

### 3.1 Demographic Characteristics and Laboratory Findings of Restless Legs Syndrome Patients and Healthy Controls

A total of 49 patients with restless leg syndrome (RLS) and 26 healthy individuals were recruited for this study and the detailed characteristics of all participants were presented in Table 1. Age and gender differences between RLS patients and healthy controls are not statistically significant (*p* > 0.05). The analysis of hematological parameters revealed that there was no significant difference in the levels of WBC, RBC, HB, HCT, platelets, Neu, Lym, and Mon between healthy controls and patients with RLS (*p* > 0.05). Additionally, we did not observe any significant difference in the blood parameters, such as ferritin, iron, and TIBC, between healthy controls and RLS patients (*p* > 0.05). We analyzed 4 main electrolytes, including Na, K, Ca, Mg in healthy controls and RLS patients and our results showed no statistically significant differences in these parameters (*p* > 0.05). Our biochemical tests revealed that there was no discernible difference between the serum samples from RLS patients and healthy individuals in terms of urea, creatinine, and AST levels (*p* > 0.05). Further, serum ALT levels were observed to be greater in RLS patients compared to healthy controls (*p* = 0.033).

**TABLE 1:** Demographic characteristics and laboratory findings of restless legs syndrome patients and healthy controls.

Variables	HC	RLS	Normal range	*p* Value
Gender (male/female)	5 / 21	14/35		0.376
Age	46.92 ± 12.68	52.28 ± 15.37		0.132
Lab parameters				
WBC, 10^3^/µL	7.30 ± 2.27	6.83 ± 1.49	4–10	0.529
RBC, 10^6^/µL	4.67 ± 0.45	4.66 ± 0.51	3.5–5	0.730
HB, g/dL	13.26 ± 1.27	12.94 ± 1.83	11–15	0.551
HCT, %	40.92 ± 3.51	39.13 ± 7.06	37–47	0.350
Platelet, 10^3^/µL	268.03 ± 63.32	260.39 ± 84.16	151–387	0.824
Neu, %	56.25 ± 12.54	55.5 ± 7.39	50–70	0.497
Neu, 10^3^/µL	3.98 ± 1.46	3.77 ± 0.98	3.5–5	0.656
Lym, %	33.15 ± 6.84	34.48 ± 8.64	20–40	0.582
Lym, 10^3^/µL	2.33 ± 0.70	2.37 ± 0.73	0.8–4	0.680
MON, %	9.34 ± 11.09	8.6 ± 10.81	3–12	0.867
MON, 10^3^/µL	0.6 ± 0.8	0.59 ± 0.83	0.12–1.2	0.529
Ferritin, ng/mL	41.79 ± 24.72	37.93 ± 34.01	4.63–204	0.201
Iron, µg/dL	74.23 ± 31.81	70.81 ± 31.24	60–180	0.616
TIBC, µg/dL	277.69 ± 92.18	312.67 ± 74.79	155–355	0.098
Na, mmol/L	142.88 ± 11.79	139.51 ± 2.25	136–146	0.330
K, mmol/L	4.39 ± 0.37	4.42 ± 0.39	3.5–5.1	0.828
Mg, mg/dL	1.96 ± 0.09	2.01 ± 0.43	15–38	0.846
Ca, mg/dL	9.57 ± 0.31	9.63 ± 0.5	8.8–10.7	0.596
CREA, mg/dL	0.75 ± 0.15	0.77 ± 0.20	0.51–0.95	0.942
Urea, mg/dL	30.50 ± 16.42	29.22 ± 11.34	17–43	0.824
ALT, U/L	17.88 ± 8.43	20.16 ± 6.47	0–35	**0.033**
AST, U/L	18.65 ± 5.34	23.93 ± 28.58	0–35	0.458

*Note*: The Mann–Whitney *U* test was used to examine continuous data, which were then displayed as mean ± standard deviation. Analyzed using the chi‐square test, categorical data were displayed as numbers. Statistically significant *p* values were denoted as bold and were less than 0.05.

Abbreviations: WBC: white blood cells, RBC: red blood cells, HGB: hemoglobin, HCT: hematocrit, NEU: neutrophil, LYM: lymphocyte, MON: monocytes, TIBC: total iron binding capacity, NA: sodium, K, potassium, Mg: magnesium, Ca: calcium, ALT: alanine aminotransferase, AST: aspartate aminotransferase, CREA: creatinine.

**TABLE 2:** The comparison of the quality of life and sleep and the depression in patients with restless legs syndrome and healthy controls.

	Healthy Controls (*n* = 26)	RLS patients (*n* = 49)	*p* Value
SF‐12 subscales			
PCS	48.86 ± 7.13	39.12 ± 11.50	**<0.001**
MCS	46.18 ± 10.52	39.07 ± 9.89	**0.002**
PSQI			
Global PSQI score	4.19 ± 2.31	12.57 ± 4.21	**<0.001**
Subjective sleep quality	0.92 ± 0.74	1.83 ± 0.87	**<0.001**
Sleep latency	1.15 ± 0.96	2.59 ± 0.53	**<0.001**
Sleep duration	0.23 ± 0.65	1.81 ± 0.97	**<0.001**
Sleep efficiency	0.19 ± 0.49	2.04 ± 1.25	**<0.001**
Sleep disturbances	1.00 ± 0.48	1.61 ± 0.63	**<0.001**
Use of sleep medication	0.15 ± 0.36	0.91 ± 0.88	**<0.001**
Daytime dysfunction	0.53 ± 0.64	1.75 ± 0.82	**<0.001**
BDI scores	9.34 ± 8.09	17.48 ± 15.01	**0.027**

*Note*: The Mann–Whitney *U* test was used to examine continuous data, which were then displayed as mean ± standard deviation. Any *p* values that were statistically significant (*p* < 0.05) were highlighted in bold.

Abbreviations: RLS: restless leg syndrome, PCS: physical component summary, MCS: mental component summary, PSQI: Pittsburgh Sleep Quality Index, BDI: mean score of the Beck Depression Inventory.

### 3.2 Assessment of the Quality of Life and Sleep and the Depression in Patients With Restless Legs Syndrome

We assessed the quality of life and sleep and the depression in patients with RLS using the SF‐12 quality of life scale, the PSQI, and the BDI, and the results are shown in Table 2. Our examination of quality of life revealed that the subdomain scores of the SF‐12 quality of life scale were notably higher in healthy individuals compared to patients with RLS. Furthermore, our sleep quality assessment unveiled that PSQI scores in RLS patients were markedly higher than those in the healthy controls. Our assessment of the severity of depressive symptoms revealed that the BDI scores were higher in patients with RLS compared to healthy individuals.

**TABLE 3:** Relationship of ATG3 and ATG5 with quality of life and sleep, the depression and laboratory markers in patients with restless legs syndrome.

	ATG3		ATG5		Ferritin		Iron		TIBC	
	*r*	*p*	*r*	*p*	*r*	*p*	*r*	*p*	*r*	*p*
RLS score	**0.295**	**0.01**	**0.370**	**0.001**	−0.250	0.034	−0.079	0.499	**0.256**	**0.026**
SF‐PCS12	−0.199	0.088	**−0.271**	**0.02**	0.127	0.289	0.030	0.800	**−0.248**	**0.032**
SF‐MCS12	**−0.229**	**0.048**	**−0.301**	**0.01**	0.163	0.170	0.003	0.977	−0.122	0.296
Global PSQI score	**0.334**	**0.003**	0.179	0.129	−0.103	0.388	−0.059	0.618	0.171	0.143
Subjective sleep quality	**0.292**	**0.011**	0.019	0.875	−0.085	0.478	0.017	0.886	0.077	0.510
Sleep latency	0.179	0.125	0.092	0.437	−0.159	0.182	−0.013	0.913	0.181	0119
Sleep duration	**0.270**	**0.019**	0.042	0.722	−0.118	0.325	−0.111	0.345	0.135	0.247
Sleep efficiency	**0.240**	**0.038**	0.220	0.061	−0.021	0.861	−0.185	0.112	0.159	0.172
Sleep disturbances	**0.297**	**0.01**	0.216	0.066	−0.122	0.309	0.084	0.473	0.102	0.383
Use of sleep medication	0.179	0.124	0.207	0.079	0.061	0.613	0.044	0.709	0.068	0.561
Daytime dysfunction	**0.289**	**0.012**	0.199	0.092	−0.038	0.748	0.044	0.709	0.157	0.178
BDI scores	0.086	0.462	**0.263**	**0.024**	−0.125	0.296	0.028	0.809	0.177	0.129
Ferritin	0.054	0.655	0.173	0.152			**0.376**	**0.001**	−**0.599**	**<0.001**
Iron	−0.018	0.879	−0.023	0.846	**0.376**	**0.001**			−**0.657**	**<0.001**
TIBC	0.103	0.377	0.056	0.637	−**0.599**	**<0.001**	−**0.657**	**<0.001**		

*Note*: The association between autophagy‐related proteins and quality of life and sleep, the depression and laboratory markers were examined using Spearman's rho test. Any *p* values that were statistically significant (*p* < 0.05) were highlighted in bold.

Abbreviations: ATG3: autophagy‐related gene 3, ATG5: autophagy‐related gene 5, RLS: restless leg syndrome, TIBC: total iron binding capacity, SF‐PCS12: Short Form 12 Physical Component Summary Score, SF‐MCS12: Short Form 12 Mental Component Summary Score, PSQI: Pittsburgh Sleep Quality Index, BDI: mean score of the Beck Depression Inventory.

### 3.3 Expression Levels of Autophagy Related Proteins in Patients With Restless Legs Syndrome

**FIGURE 1:**
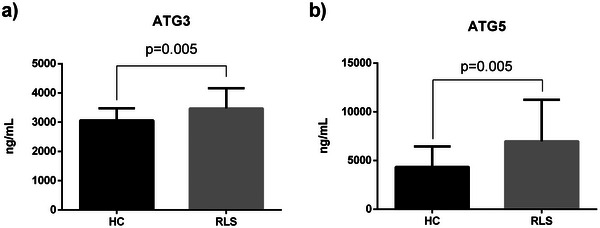
Comparison of Serum ATG3 (a) and ATG5 (b) expressions levels in patients with RLS. RLS: restless leg syndrome, ATG3: autophagy‐related gene 3, ATG5: autophagy‐related gene 5.

In order to ascertain whether there were disparities in ATG3 and ATG5 levels between the patient and control groups, we assessed the protein expressions of ATG3 and ATG5 in serum samples and depicted the results in Figure 1. The results of our analysis showed a substantial increase in the expressions of ATG3 and ATG5 in patients with RLS compared to healthy controls (*p* < 0.005, *p* < 0.006, respectively) (Figure 1a, b).

### 3.4 Association of Autophagy‐Related Proteins With the Pathogenesis of Restless Legs Syndrome

We analyzed the association of serum autophagy‐related protein levels with the quality of life and sleep, the depression and laboratory markers in patients with RLS and healthy individuals. Our analyses demonstrated that the severity scores of RLS are positively correlated with both ATG3 and ATG5 levels, as illustrated in Table 3. We observed that serum ATG3 levels were negatively correlated with SF‐MCS12. Moreover, we found that serum ATG5 levels were negatively correlated with subscale scores of the SF‐12, including SF‐MCS12 and SF‐PCS12. Our correlation analyses demonstrated that serum ATG3 levels were significantly correlated with sleep quality. Subscale scores of the PSQI, such as subjective sleep quality, total sleep duration, sleep efficiency, sleep disturbances, and daytime dysfunction, were positively correlated with serum ATG3 expressions. Furthermore, there was a positive correlation found between depression levels and serum ATG5 concentrations.

### 3.5 Diagnostic Importance of Autophagy‐Related Proteins for Patients With Restless Legs Syndrome

**FIGURE 2:**
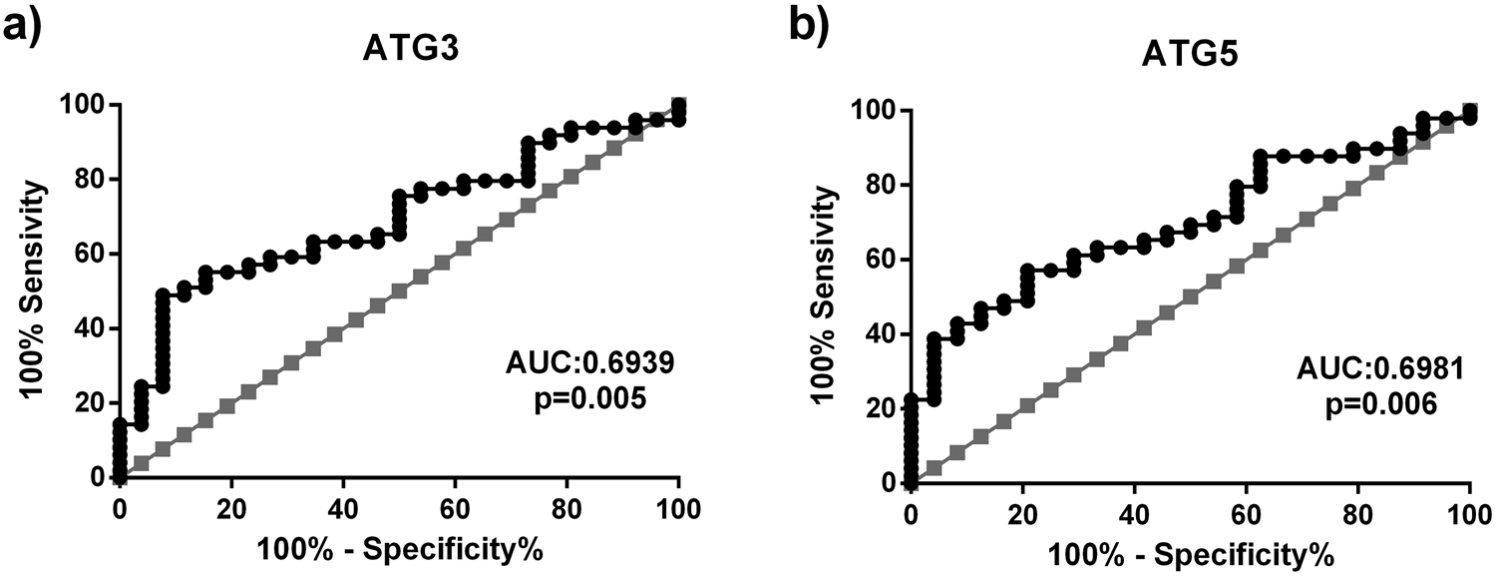
The evaluation of potential predictive values of ATG3 (a) and ATG5 (b) in patients with RLS using ROC curve analyses. RLS: restless leg syndrome, ATG3: autophagy‐related gene 3, ATG5: autophagy‐related gene 5, ROC: receiver‐operating characteristic curve.

We conducted ROC curve analyses to determine whether variations in ATG3 and ATG5 expressions could be used as biomarkers to predict the occurrence of RLS and presented the results in Figure 2 and Table 4. Our ROC curve analyses revealed that blood ATG3 expressions were able to distinguish between patients with RLS and healthy controls (*p* = 0.005). With a sensitivity of 63.3% and a specificity of 61.5%, the optimal ATG3 cut‐off value for predicting the presence of RLS was 3146.5 ng/mL. Moreover, our data showed that blood ATG5 expressions might differentiate between patients with RLS and healthy controls (*p* = 0.006). We found that the ATG5 expressions greater than 4732.5 ng/mL with a sensitivity of 63.3% and specificity of 62.5% reliably predict the presence of RLS.

**TABLE 4:** The receiver operating characteristic curve analyze results and optimal cut‐off levels of ATG3 and ATG5 in patients with restless legs syndrome.

Variables	AUC (95% CI)	Sensitivity %	Specificity %	Cut‐off	*p* value
ATG3	0.6939 (0.5141–0.8136)	63.3	61.5	3146.5 ng/mL	**0.005**
ATG5	0.6981 (0.5783–0.8180)	63.3	62.5	4732.5 ng/mL	**0.006**

Any *p* values that were statistically significant (*p* < 0.05) were highlighted in bold.

Abbreviation: AUC: area under the receiver operating characteristic curve, CI: confidence intervals, RLS: restless leg syndrome, ATG3: autophagy‐related gene 3, ATG5: autophagy‐related gene 5, ROC: receiver‐operating characteristic curve.

## 4 Discussion

Restless legs syndrome is a neurological disorder that causes a considerable socioeconomic impact, and its molecular pathophysiology is still unknown (Gossard et al. 2021). Thus, scientists all over the world focus largely on not only trying to understand the molecular pathophysiology of RLS but also finding new biomarkers and therapeutic targets for RLS management to enhance the clinical results of the illness (Alacam Koksal et al. 2023; Laleli Koc et al. 2023). Existing studies indicate that abnormalities in dopaminergic system function and reduced iron levels in the central nervous system are significant factors in the onset and development of RLS (Allen 2007; Weinstock, Walters, and Paueksakon 2012). Additionally, it has been reported that the iron storage and dopaminergic system are associated with autophagic mechanisms (Inoue et al. 2017; Krishan et al. 2015; Munoz et al. 2012; Nanayakkara, Di Michiel, and Yee 2023). Despite advancements in understanding the potential diagnostic and therapeutic advantages of autophagic proteins in many human illnesses, including Parkinson's disease, Alzheimer's disease, Multiple Sclerosis, and Coronavirus disease 2019, the precise role of autophagy‐related proteins in the development of RLS remains incompletely understood (Changotra et al. 2022; Cho et al. 2019; Joodi Khanghah et al. 2020; Okuyan et al. 2021).

The current study is the first to examine the potential involvement of autophagy‐related proteins in patients with RLS. We revealed that ATG3 and ATG5 expressions in serum samples were upregulated in patients with RLS and the severity of the disease was correlated with the increased expressions of ATG3 and ATG5. Furthermore, the correlation analyses indicated that ATG3 was substantially correlated with the quality of sleep, whereas ATG5 was correlated with the quality of life and depression status. Additionally, the correlation analyses demonstrated that ATG3 was significantly correlated with the quality of sleep, while ATG5 was associated with the quality of life and depression status. Our ROC studies showed that ATG3 and ATG5 have the potential to be useful prognostic or diagnostic indicators in the clinical management of RLS, with good sensitivity and specificity.

ATG3, a regulator enzyme, plays an important role in the LC3 lipidation process, which is required for autophagy formation (da Silva Lima et al. 2022). A limited number of studies has shown that ATG3 was dysregulated in some clinical disorders, including cerebral ischemia/reperfusion injury, gastric and colon cancer (da Silva Lima et al. 2022; Huang et al. 2019; Peng et al. 2021). The findings of these studies offer substantial evidence on the involvement of ATG3 in several pathophysiological processes, such as damage, inflammation, proliferation, invasion, and mitochondrial function (da Silva Lima et al. 2022; Huang et al. 2019; Peng et al. 2021). Here, we demonstrate for the first time that ATG3 expressions were elevated in RLS patients and upregulated ATG3 levels were associated with the severity of the disease. In addition, these upregulated expressions were associated with the quality of life and sleep in RLS patients. Additionally, our ROC analysis indicated that ATG3 may be important for diagnosing RLS and managing it clinically with good sensitivity and specificity.

ATG5, another regulator protein of autophagic mechanism, exhibited dysregulation in a number of clinical conditions, such as allergic rhinitis, Alzheimer's disease, and multiple sclerosis (Changotra et al. 2022; Cho et al. 2019; Joodi Khanghah et al. 2020; Zheng and Wang 2023). In previous research, Joodi Khanghah et al. (2020) reported that serum ATG5 expressions could have diagnostic and prognostic value for multiple sclerosis. In a recent research, Cho et al. (2019) unveiled that plasma ATG5 expressions were elevated in Alzheimer's disease patients. Their findings suggested that ATG5 is associated with the pathogenesis of Alzheimer's disease and may serve as a biomarker (Cho et al. 2019). In our study, we revealed that ATG5 expressions were upregulated in RLS patients and the upregulated ATG5 expressions were positively correlated with the clinical severity of the disease. Further, we showed that increased ATG5 expressions were associated with the quality of life and sleep and the depression status in RLS patients. Our ROC analysis revealed that ATG5 might be utilized as diagnostic and prognostic indicators for clinical management of RLS with good sensitivity and specificity. Our data presented here suggest that both ATG3 and ATG5 reflect the clinical conditions of RLS patients. Moreover, considering the relationship of autophagy with iron deficiency and dopaminergic system (Inoue et al. 2017; Krishan et al. 2015; Munoz et al. 2012; Nanayakkara, Di Michiel, and Yee 2023), we may speculate that the dysfunction of these mechanisms in RLS patients stimulates autophagy‐related protein expressions as a compensatory mechanism for RLS.

In addition, we assessed the routine blood parameters in RLS patients in the current research. Previous studies have drawn attention to the inconsistent results of routine laboratory parameter between healthy controls and RLS patients (Alacam Koksal et al. 2023; Olgun et al. 2019; Tufekci and Kara 2021). For instance, Olgun Yazar et al. (2019) demonstrated a reduction in iron and ferritin levels in patients with RLS when compared to healthy controls. In another study, it has been shown that there is no notable difference in iron and ferritin levels between healthy controls and RLS patients (Alacam Koksal et al. 2023). In the present study, we found no difference in iron and ferritin concentrations among the groups. It is not unexpected that there is no disparity in the serum levels of ferritin, iron, and hemoglobin between patients with RLS and the healthy controls. In fact, observed variations appear to be present solely in the cerebrospinal fluid. Therefore, impaired iron transport from the bloodstream to the central nervous system appears to be the underlying cause of low brain iron levels in patients with RLS (Mizuno et al. 2005). Furthermore, iron and ferritin values ​​measured in peripheral blood are not good indicators of intracellular iron levels in the central nervous system because of their important roles in enzymatic functions. These data emphasize that RLS is linked to the central nervous system rather than the peripheral system.

Even though we present significant data regarding the role of autophagy in the pathophysiology of RLS, there are a few limitations to our study. The results we have presented here should be validated by multi‐center and large‐scale research, as the sample size of the study population was relatively small. Further, how pharmaceutical treatment and iron supplements changed autophagy‐related protein expressions in the RLS patients under observation at the time of sample collecting is unknown. Another limitation of the current study is the lack of knowledge regarding the variation in autophagy‐related protein expressions prior to and following treatment.

## 5 Conclusions

Our data presented here for the first time suggest that autophagy may have a significant impact on the development of RLS. Serum levels of autophagy‐related proteins were increased in patients with RLS and upregulated ATG3 and ATG5 expressions were relationship with the severity of the disease. The present results indicated that ATG3 was substantially correlated with the quality of sleep, whereas ATG5 was correlated with the quality of life and depression status. Moreover, our ROC analyses demonstrated that ATG3 and ATG5 have the potential to serve as reliable prognostic or diagnostic markers in the clinical management of RLS, exhibiting good sensitivity and specificity. Further investigation into the role of autophagy in the molecular mechanisms of RLS might provide valuable insights into the underlying causes of RLS.

## Author Contributions

**Esen Çiçekli**: conceptualization, methodology, investigation, formal analysis, supervision, writing–original draft, writing–review and editing, resources. **Hamza Malik Okuyan**: conceptualization, methodology, investigation, formal analysis, supervision, resources, writing–review and editing, writing–original draft, visualization. **Canan Birimoğlu Okuyan**: investigation, methodology, supervision, visualization, writing–original draft, writing–review and editing. **Şeyda Öznur Ayçiçek**: methodology, writing–original draft, writing–review and editing, formal analysis, investigation. **Dilcan Kotan**: investigation, supervision, writing–review and editing, writing–original draft, resources, visualization, methodology.

## Conflicts of Interest

The authors declare that the research was conducted in the absence of any commercial or financial relationships that could be construed as potential conflicts of interest.

### Peer Review

The peer review history for this article is available at https://publons.com/publon/10.1002/brb3.70172.

## Data Availability

The data sets used and/or analyzed during this study are available from the corresponding author (HMO) upon reasonable request.
